# Implementation of a multi-level intervention in underserved communities: Prevention RCT in the Cherokee Nation

**DOI:** 10.1186/1748-5908-10-S1-A54

**Published:** 2015-08-20

**Authors:** Kelli A Komro, Misty L Boyd, Melvin D Livingston, Terrence Kominsky, Dallas Pettigrew, Brady Garrett, Alexander C Wagenaar

**Affiliations:** 1 Department of Health Outcomes and Policy, College of Medicine, University of Florida, Gainesville, FL 32608, USA; 2 Institute for Child Health Policy, University of Florida, Gainesville, FL 32608, USA; 3 Cherokee Nation Behavioral Health, Tahlequah, OK 74465, USA

## 

Despite advances in prevention science in recent decades, the U.S. continues to struggle with significant alcohol-related risks and consequences among youths, especially among vulnerable rural and American Indian communities. The Prevention Trial in the Cherokee Nation is a partnership between prevention/implementation scientists and Cherokee Nation Behavioral Health to implement and evaluate an integrated multi-level intervention designed to prevent underage drinking and associated negative health outcomes among American Indian and other youths living in rural high-risk underserved communities. The primary partnership is augmented with partnerships with Oklahoma public school districts, the Oklahoma Department of Human Services, and a local nonprofit organization. The intervention builds directly on results of multiple previous RCTs of two conceptually distinct approaches. The first is an updated version of an evidence-based community environmental change intervention, and the second is our newly developed population-wide implementation of screening, brief intervention and referral to treatment delivered within high schools by school service providers hired through the Department of Human Services. Six key research design elements optimize causal inference and experimental evaluation of effects of intervention implementation, including purposive selection of towns, random assignment of towns to study condition, controlled interrupted time-series, nested cohorts as well as repeated cross-sectional observations, a factorial design crossing two conceptually distinct interventions, and multiple comparison groups. Interim results indicate a high uptake of intervention implementation with some variation by community, as well as trends toward decreased alcohol use among youths in the intervention conditions compared to the control condition. The trial demonstrates the effectiveness of scientists building strong partnerships for implementation of evidence-based strategies within underserved communities. Our collaborative partnerships also demonstrate it is feasible to implement a rigorous scientific trial while incorporating community-based participatory research methods.

Research was supported by the National Institute on Alcohol Abuse and Alcoholism of the National Institutes of Health under Award Number R01AA020695.

